# The Use of Remote Monitoring Technologies: A Review of Recent Regulatory Scientific Advices, Qualification Opinions, and Qualification Advices Issued by the European Medicines Agency

**DOI:** 10.3389/fmed.2021.619513

**Published:** 2021-07-01

**Authors:** Marieke J. H. J. Dekker, Pieter Stolk, Anna M. G. Pasmooij

**Affiliations:** ^1^Medicines Evaluation Board, Utrecht, Netherlands; ^2^Lygature, Utrecht, Netherlands

**Keywords:** remote monitoring devices, European Medicine Agency, scientific advices, qualification advices, qualification opinions

## Abstract

**Aims:** Recently, the use of novel remote monitoring technologies (RMTs) in trials has gained much interest. To facilitate regulatory learning, we evaluated qualification opinions (QOs) and advices (QAs) and scientific advices (SAs) of the Committee for Medicinal Products for Human Use (CHMP) to gain insight in the types of devices that are intended to be used in clinical trials for supporting/submitting application for obtaining marketing authorization (registration trials) and the main recommendations of the CHMP.

**Methods:** QOs, QAs, and SAs of the CHMP that assessed RMTs between 2013 and 2019 were eligible for our study. The following information was extracted from the documents: year of advice/opinion, device and endpoints used, type of endpoint (primary, secondary, exploratory, or safety), and main recommendations of the CHMP.

**Results:** In total two QOs, four QAs, and 59 SAs were included in our study (total of SAs between 2013 and 2019 = 4,054). In the SAs, accelerometers to measure activity and/or sleep parameters (*n* = 31) were the most frequently used devices, followed by mobile applications (*n* = 6) and glucose monitoring devices (*n* = 6). Usually, these measures were proposed as secondary or exploratory endpoints (*n* = 32). The main recommendations of the CHMP were related to relevance of the (novel) outcome measure; validation; precision, accuracy, sensitivity, and specificity; compliance; sampling interval; and data handling and privacy.

**Conclusions:** Although there was a trend toward an increased use over time, the use of RMTs in registration trials is still relatively rare. In the absence of formal European regulatory guidance on mHealth technologies, insight in the main recommendations of the CHMP may stimulate the use of novel RMTs in a regulatory context.

## Introduction

In recent years, remote monitoring technologies (RMTs) have rapidly evolved and gained increasing interest of health technology industry, clinicians, medicine developers, and regulators (1). Several public–private initiatives have emerged that provide platforms for collaborations between patient, clinical, and research communities as well as mHealth companies to promote new developments, provide guidance, and take patient and consumers views into account. The Duke Margolis Center, for example, convened a working group of experts, including the Food and Drug Administration (FDA) and released a set of recommendations on how to promote efficient and ethical research-capable technologies such as mHealth apps and wearables for real-world evidence generation (2).

RMTs offer new opportunities to assess novel endpoints or possibly better ways to measure existing endpoints. With these technologies, endpoints can be assessed in the home environment, which has several advantages. For example, measurements are less time point dependent and are able to capture fluctuations in disease activity and activity patterns, such as differences between weekdays and weekends. Next to this, less visits to clinics are necessary, which may decrease participation burden and promote participation. Furthermore, endpoints can be assessed with high frequency, which improves data completeness and sensitivity. Lastly, RMTs offer objective ways for real-time outcome measurement and may reduce white coat effects.

From a regulatory perspective, it is important that novel endpoints are reliable, accurate, sensitive to change, and validated for purpose of use. The Clinical Trials Transformation Initiative (CTTI), a collaboration between pharma companies, academics, and the FDA, issued recommendations on the development of novel endpoints generated by mobile technology for use in clinical trials (3). These recommendations focus on optimizing novel endpoint selection as well as practical approaches to the novel endpoint development process. In Europe, the Heads of Medicines Agency (HMA)/European Medicines Agency (EMA) Task Force on Big Data has released a subgroup report on “Social Media and M-Health Data” that explored social media sites and mHealth technologies that could be valuable to support medicine regulation decision-making and its main challenges in using these data for regulatory purposes (1). Furthermore, as of June 2020, the EMA published *Questions and Answers (Q&A): Qualification of digital technology-based methodologies to support approval of medicinal products* (4). This Q&A document does not contain comprehensive guidance but reflects the EMA's current experience, and further considerations may be added as the EMA's experience increases. However, it does provide some important recommendations to consider for successful qualification of digital technology-based methodologies.

In the European regulatory context, the EMA provides three different procedures for device manufacturers and pharma companies to obtain feedback from the Committee for Medicinal Products for Human Use (CHMP). First, a qualification opinion (QO) issues an opinion on the acceptability of a specific use of a novel methodology in the context of research and development (5). This opinion is publically available on the EMA website and based on the assessment of data submitted to the EMA. The CHMP can also issue an advice on protocols and methods that are intended to develop a novel method with the aim of moving towards qualification (5). Additionally, the CHMP can provide medicine developers advice on study protocols, including endpoint selection, of a medicinal product with the aim of marketing approval (6). Both qualification advices (QAs) and scientific advices (SAs) are confidential and only provided to applicants.

The aim of the current study was to evaluate the scope and types of RMTs that are currently intended to be used in clinical trials for supporting/submitting application for obtaining marketing authorization (registration trials). Furthermore, we systematically collected the main recommendations and attention points of the CHMP concerning endpoints that are generated by mHealth technologies by evaluating the QOs, QAs, and SAs of the CHMP between 2013 and 2019 that covered RMTs.

## Methods

SAs, QAs, and QOs of the CHMP of the EMA were evaluated on the use of RMTs in registration trials that were issued between 2013, and 2019. QAs and QOs that assessed novel endpoints or improved established endpoints measured by mHealth technologies that can be used in a remote setting were included in our study. Likewise, SAs that covered studies using endpoints measured by RMTs were eligible for our study (inclusion criteria). We excluded advices and opinions that covered technologies that were solely used for the following: to document questionnaires and patient-reported outcome measures, for the delivery or administration of medicinal products, in animal studies or other preclinical studies, for therapeutic purposes, and non-electronic devices (exclusion criteria). In case more than one SA covered the same medicinal product and indication, only the first advice was included in our study.

QOs on novel methodologies for medicine development were accessed through the EMA website (https://www.ema.europa.eu/en/human-regulatory/research-development/scientific-advice-protocol-assistance/qualification-novel-methodologies-medicine-development) and QAs and SAs through the internal EMA database that contains these confidential documents. First, we identified potentially relevant documents in this database using the search terms “device” and “electronic.” Next, we additionally searched the database using the following search terms that were based on findings of the first step: actimeter, actigraphy, actimetry, wearable, accelerometer, GPS, smartphone, and six specific names of commercial RMTs. All QAs and SAs that were identified using these search terms were carefully read to determine whether or not they met the inclusion and exclusion criteria. Likewise, only QOs that met the inclusion and exclusion criteria were included.

From the QOs and QAs, we extracted the following information: the device used and novel outcome measure(s), date, and main recommendations of the CHMP. Likewise, we collected information on the device(s) used and outcome measure(s), date, type of endpoint(s) (primary, secondary, or exploratory), and main recommendations of the CHMP of the SAs. In case an endpoint was proposed by the applicant as a primary (or secondary) endpoint, but this was not endorsed by the CHMP, the opinion of the CHMP was adopted. Since both QAs and SAs are confidential, we could not provide any information that could lead back to a specific medicinal product (SAs) or a novel endpoint (QAs). Therefore, results from these documents are presented at an aggregated level. This approach was approved by the legal departments of the Dutch Medicines Evaluation Board (MEB) and EMA.

## Results

**Figure 1:**
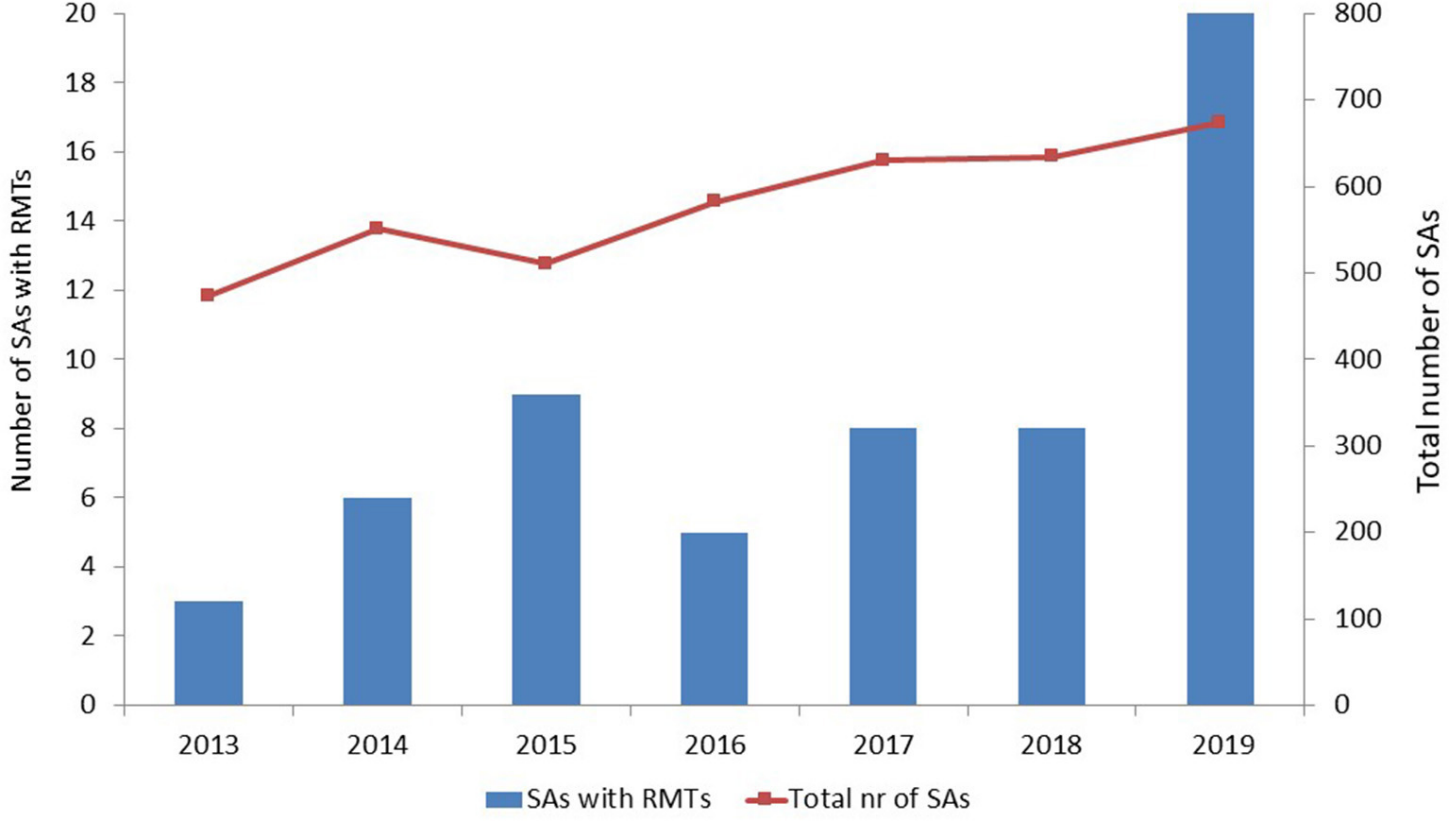
Number of scientific advices that included remote monitoring technologies and total number of scientific advices per year. SAs, scientific advices; RMTs, remote monitoring technologies.

Two QOs [stride velocity 95th percentile in Duchenne muscular dystrophy (7) and proactive in COPD (8)], four QAs, and 59 SAs met our inclusion and exclusion criteria. The number of SAs that included RMTs tended to increase over time, especially in 2019; however, the total number of SAs that the EMA issued also increased over time (Figure 1).

In a majority of the 59 SAs, accelerometers were proposed (*n* = 31) to monitor different activity and/or sleep parameters (Table 1). Other common devices included remote electronic peak flow, glucose, blood pressure, and heart rhythm measurement devices. In six QAs, mobile applications (apps) were considered for outcome measurement. These included apps to perform active tests (*n* = 2) and more complex mobile apps that combined both active and passive monitoring (*n* = 4). In a majority of cases, these mobile apps were proposed for measuring disease activity in neurologic or psychiatric disease areas (*n* = 5).

**Table 1:** Overview of remote monitoring devices and outcome measures of the scientific advices.

**Device**	**Type of outcome measure(s)**	**Number of scientific advices**
Accelerometer^*^	Activity measure(s)	17
Accelerometer^*^	Sleep measure(s)	9
Accelerometer^*^	Activity and sleep measures	3
Accelerometer^*^	Sleep and itch patterns	2
Electronic peak flow meter	Different lung function measures	3
Blood pressure measurement device	Different blood pressure measures	3
Heart rhythm and ECG measurement devices	ECG parameters and/or arrhythmias	3
(Continuous) glucose monitoring device	Glucose control measures^#^	6
Cough monitor	Cough frequency	3
App (active tests)	Measures of cognitive function	2
App (active and passive monitoring)	Different measures of disease activity	4
Not specified^∧^		4

* *Different types of devices that contain accelerometers were combined*,

# *including ketone measurement (n = 1)*,

∧ *included are the following technologies: actigraphy without specification of outcome of interest (n = 1), “mobile wearable device to measure physiological parameters” (n = 1), “mobile technology (app) and telemedicine capabilities” to perform virtual clinics and monitor safety and efficacy (n = 1), and technology to conduct study visits remotely (n = 1)*.

**Figure 2:**
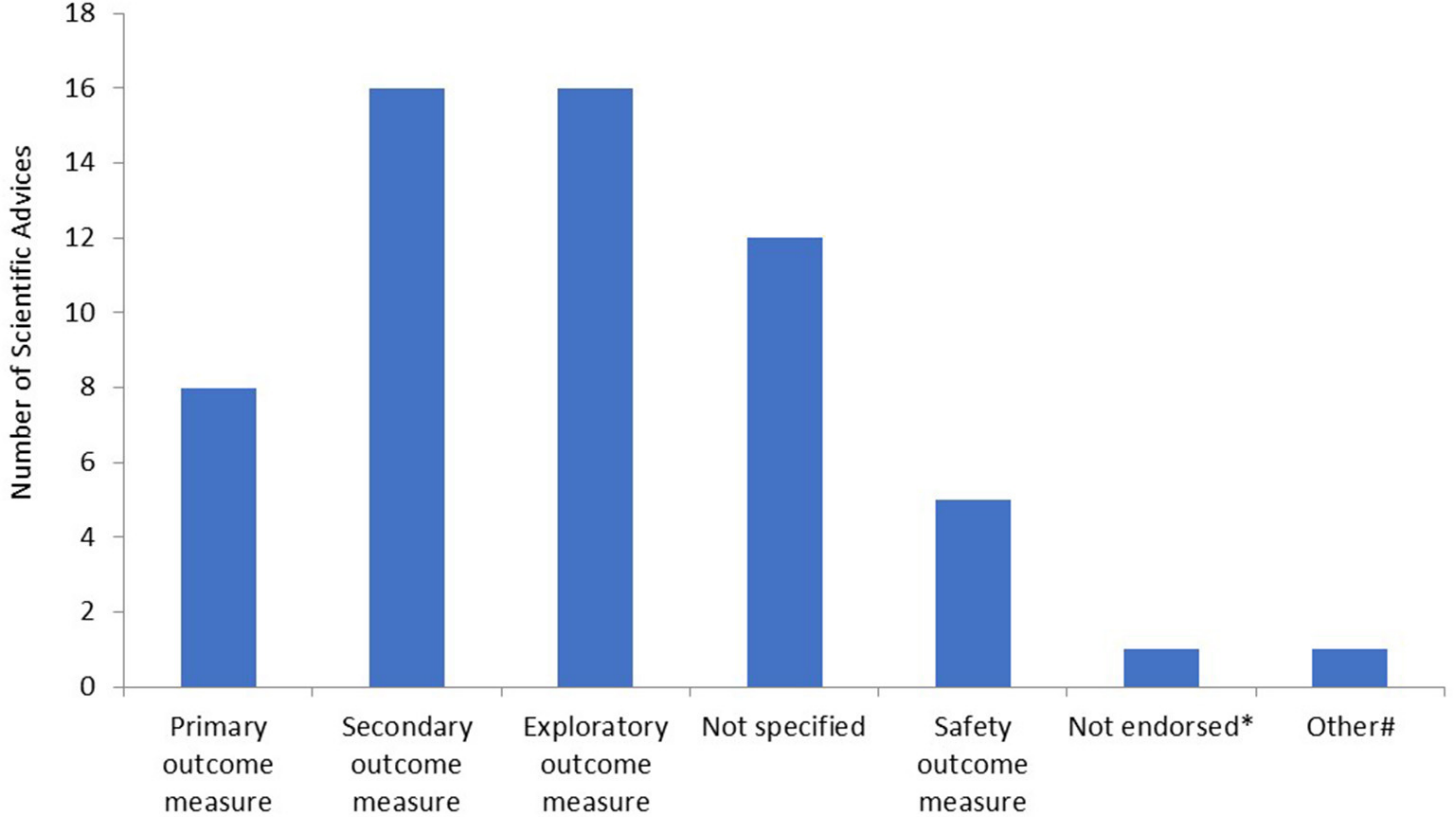
Type of endpoints as measured by remote monitoring technologies. *In this scientific advice (SA), the remote monitoring technology (RMT) was not endorsed by the Committee for Medicinal Products for Human Use (CHMP) and another method for outcome measurement was proposed by the CHMP; ^#^in this SA, the RMT was used in an explorative natural history study.

Subsequently, we determined for what type of endpoints the RMTs were proposed in the SAs. The majority of RMTs were proposed as secondary or exploratory endpoints (Figure 2). Furthermore, in 12 SAs, it was not entirely clear for what type of endpoint the applicant intended to use the outcome measure. In the majority of these cases, there was no clear distinction made between secondary or exploratory endpoints. In eight SAs, the measurement was accepted by the CHMP as a primary or co-primary endpoint. Furthermore, in one SA, the RMT was not endorsed by the CHMP, and an alternative method to assess the outcome measure was proposed. Lastly, in one SA, the RMT was intended to be used for outcome measurement in an explorative natural history study. Safety measures included the following: ketone or hypoglycemia measurement (*n* = 2) and remote measurement of QT/QTc intervals or detection of arrhythmias (*n* = 3).

The main questions and recommendations of the CHMP for applicants of the QOs, QAs, and SAs are summarized in Table 2. The main questions and concerns of the CHMP were related to (1) the relevance of the (novel) outcome measure for the disease; (2) validation of the novel outcome measure; (3) precision, accuracy, sensitivity, and specificity of the novel endpoint; (4) compliance and handling of missing values; (5) sampling interval; and (6) data handling, accessibility, and privacy. In case the novel endpoint was expected to be an improved measure compared to the established outcome measure, not all recommendations of Table 2 do fully apply. This was acknowledged by the CHMP. Additionally, applicants frequently asked the CHMP questions relating to the medical device regulation. However, this is not the remit of the CHMP, and applicants were advised to direct these questions to a notified body.

**Table 2:** Main concerns and questions of the CHMP for the applicants.

**Main recommendations**
**A. Relevance of the (novel) outcome measure as assessed by the remote monitoring technology**
Is the outcome measure relevant for the disease of interest? Does the device measure all aspects or symptoms of a specific disease? Does the device measure all aspects of a specific function? To what extent is the outcome measure only influenced by the disease activity of interest? How does the outcome measure relate to the CHMP guideline of the corresponding disease? In case of sensor data: to what extent is the body part to which the sensor is attached reflective of the symptoms of the disease or condition of interest?
**B. Validation of the novel outcome measure as assessed by the remote monitoring technology**
Is the outcome measure correlated with hard endpoints? (morbidity/mortality) Is the outcome measure correlated with relevant outcome measures for patients? [Quality of life (QOL) and patient-reported outcome measures (PROMs)] Is the outcome measure correlated with the established/golden standard clinical tests and/or PROMs of the disease of interest? In case several established outcome measures exist that measure different symptoms or aspects of a disease: to what extent does the novel outcome correlate with all these different endpoints? (NB relevance of an outcome measure can depend on the intended treatment effect) Is a change in the novel endpoint correlated with a change in final endpoints or (other) outcomes that matter to patients? (QOL or PROMs) What is the external validity? In how many patients/persons has the device been tested? Is the minimal clinically important change determined? If yes: is this studied prospectively? Is the minimal clinically important change determined for the different diseases, subgroups and clinical stages of interest?
**C. Precision, accuracy, sensitivity, and specificity**
To what extent does the novel endpoint predict the established endpoint(s) with enough precision? What is the internal validity? (same value in stable patients) Are there systematic errors in measurements in specific subgroups? (e.g., overestimation of walking speed in more severe multiple sclerosis patients due to ataxia) In case several outcome measures are available for a device: what outcome measure is chosen and why? What is the effect of outliers and was this taken into account? To what extent can the device check what the activity of the patient is during the measurement? If relevant: how is this issue handled? Is the outcome measure sensitive enough to distinguish relevant subgroups? Technical correctness: to what extent is the device capable of measuring a change that is clinically relevant?
**D. Compliance and handling of missing values**
What is the compliance? Is the compliance stable during follow-up? Is selective non-compliance an issue? (e.g., a patient does not perform tests or wears a device during periods of increased symptomatology) What measures are taken to prevent (selective) missing values? Is compliance actively stimulated? (e.g., by the use of alarms, phone calls, etc.) How are missing data handled? In case of an active test: is the test performed every day at the same time? Is a medication tracker used? (this can be of relevance in case of clear on/off states such as in Parkinson's disease) Wat is the tolerability and acceptability of the technology for patients?
**E. Sampling interval**
Is the sampling interval long enough to take day-to-day variation into account? Is the sampling interval per measurement short enough that no clinical change is expected and optimal compliance is expected? How is the sampling interval determined?
**F. Privacy and data handling**
How are data anonymized and protected? Who has access to the data? Is a risk analysis performed to guarantee optimal data security?

Some attention points of the CHMP will be illustrated. Applicants should provide information on compliance with a technology and handling of missing values. Selective non-compliance might be an issue, especially if compliance is relatively low. In case of mobile applications for instance, patients may be less likely to perform active tests when they experience more symptoms. This might partly be prevented by instructing the patient to perform the test every day at the same time, and electronic reminders such as alarms. Furthermore, the use of medication trackers might be relevant, especially for conditions with clear on/off states such as Parkinson's disease where patients can experience much more symptoms when levodopa starts to wear off (category D: compliance).

For many novel endpoints generated by RMTs, sampling intervals need to be determined. In case of “stride velocity 95th percentile in Duchenne muscular dystrophy measured by a wearable and valid device,” a recording period of 180 h per month was chosen [QO, (7)]. Argumentations were that (A) variability decreased up to a plateau after this recording period, (B) this period seemed short enough to be used as a baseline measurement and long enough to cover week-to-week variations in activity, (C) disease progression is not expected during this period, and (D) patient burden was not considered to be too strenuous (category E: sampling interval). This sampling interval was endorsed by the CHMP.

Since RMT data need to be stored and transported to the research site, data handling and privacy issues need to be addressed. In case of the QO of stride velocity 95th percentile in Duchenne muscular dystrophy, a risk analysis was conducted by the applicant and considered acceptable by the CHMP. One reason that privacy was not a big concern in this QO was that the data recorded were only motion sensors of wrist and ankles, and no private information such as GPS location or name and address could be retrieved from the wearable device and system measures (category F: privacy and data handling).

## Discussion

The present study shows that the use of RMTs in a regulatory context is still relatively rare, and the majority of RMTs were proposed for measurement of secondary or exploratory endpoints. The most commonly used RMTs are accelerometers that can evaluate both measures of activity and sleep. Other RMTs include mobile apps that track disease activity, electronic peak flow meters, continuous glucose monitoring, blood pressure and heart rhythm monitoring, and remote cough measurement devices. Most recommendations of the CHMP apply to all novel endpoints and are not specific for mHealth technologies, such as relevance of the novel endpoint for the indication of interest; validation with current golden standard and legacy endpoints; and sensitivity, specificity, accuracy, and precision of the novel endpoint. Recommendations that are more specific for RMTs include good compliance and acceptability of the novel technology and guarantee of optimal data security and privacy.

Currently, comprehensive guidance on the development of novel endpoints generated by mHealth technologies is lacking in Europe. However, the EMA recently published a Q&A on the qualification of digital technologies (4), and globally, several other initiatives in this field exist. The CTTI, a public–private partnership between the FDA, academics, and pharma companies, was created in 2007 to develop practices that will increase the quality and efficiency of clinical trials. The CTTI project “Novel Endpoints” issued recommendations for the development of a novel endpoint generated by mobile technologies, including a stepwise approach for the development process (3, 9). This approach consists of a first section that describes a pathway for selection of outcome assessment, mobile technology, and patient population as well as a second section that addresses specific development steps for a mobile technology-derived outcome assessment into an endpoint for regulatory clinical trials (9).

The importance of most of the steps of the CTTI approach is also stressed by the CHMP. One exception is the CTTI recommendation to develop a user manual. This was not explicitly recommended by the CHMP in the QOs and QAs we evaluated, possibly because this was already provided by most applicants. Furthermore, in its approach, the CTTI emphasizes the importance of patients' and caregivers' insights in the selection process of meaningful health aspects, concepts of interests, and specific measurements. Although the CHMP specifically requested information on the correlation of novel endpoints with outcome measures such as quality of life and patient-reported outcome measures (PROMs) in many advices and opinions, this specific point of attention was made less clear by the CHMP in the documents we evaluated regarding RMTs. In general, in the assessment of novel endpoints generated by RMTs, the CHMP focused on validation of the new outcome measure with a golden standard or legacy measure that are usually part of the CHMP guideline for the corresponding disease. Although patient organizations and individual patients currently participate in several boards, committees, and working parties of the EMA, including SA and *ad-hoc* expert groups (10), patients' views on selection of novel endpoints and technologies could get a more prominent place in the EMA's procedures.

Many organizations underline the importance of pre-competitive collaboration, for instance for the development of industry-wide standards for the collection and reporting of data captured by mobile technologies and algorithms used to convert the data into medically meaningful endpoints (2, 9). Several of these collaborations exist (11–14), including different Innovative Medicines Initiative (IMI) projects such as IMI WEB-RADR that developed a mobile app for adverse drug reaction reporting (15). The Duke-Margolis Center for Health Policy also recommends to create such collaborations in their mHealth action plan for real-world evidence generation that they released in collaboration with the FDA (2). They propose a learning mHealth research community, in which patient representatives, analytics tool companies, device and pharma industries, clinical societies and healthcare centers, researchers, payers, and regulators participate. In their plan, the mHealth research community should consist of four learning areas that focus on patient engagement, clinician engagement, methods and tools for using mHealth data, and defining fit-for-purpose.

In Europe, the HMA-EMA Joint Big Data Taskforce issued a subgroup report on social media and mHealth data (1). They recommended to bring relevant stakeholders together to promote the use of innovative mHealth technologies and facilitate learning of regulators on topics such as technological capability, data quality, and analytical methodologies for mHealth technologies. Next to this, they advised to liaise with medical device regulators to ensure effective regulation of mHealth devices. Furthermore, regulators could contribute to data quality by more proactively defining expectations, for instance by defining to what extent and type of validation is required for different types of mHealth data, and considering the need for specific regulatory guidance. Lastly, they recommended to explore how apps and mHealth devices might be used within pharmacovigilance and post-authorization research. Although this subgroup report was a good starting point, it did not provide specific guidance on the use of mHealth technologies in registration trials. Possibly, this might (partly) explain why our study shows that RMTs are infrequently used in the regulatory context up until 2019, despite recent technological developments.

As of June 2020, the EMA published a Q&A on the *Qualification of digital technology-based methodologies to support approval of medicinal products* (4). Although this document is not intended as comprehensive guidance, it provides some key recommendations for successful qualification. As expected, most points addressed in this Q&A were also identified by the evaluation of opinions and advices of our study, which were issued before this Q&A was available. However, some recommendations were not explicitly mentioned in our overview of the main recommendation of the CHMPA. These include (1) rationale to support the added benefit as compared to traditional methods; (2) evolution of the device throughout the validation program, what changes were made to the system, when, and their potential impact; and (3) ensuring the correct use of the technology by a best practice guide. Furthermore, the Q&A emphasizes the importance of “the context of use” as a critical reference point for regulatory assessment of any qualification application. In this context, the impact of the use of different digital technologies (e.g., bring your own device) should be discussed, and a risk management plan should be provided, including for example information on interference with other applications on the device, effect of upgrades, etc. Lastly, the Q&A contains some practical recommendations such as early interaction with EMA during the development process.

To the best of our knowledge, the current study is the first study that systematically evaluates the opinions and recommendations of the CHMP on the use of RMTs in QOs, QAs, and SAs. Next to this, our study provides insight in the current use of RMTs in a regulatory context. Limitations of our study are that we focused on RMTs that measure efficacy and safety endpoints. We excluded advices and opinions that covered technologies that were solely used to measure compliance and devices that were only used as e-diaries or questionnaires, even if these devices were used to assess efficacy or safety outcomes. Furthermore, detailed recommendations for validation of composite scores, for instance in case of mobile apps combining passive and active tests, were considered beyond the scope of this research. Next, we could have erroneously excluded relevant advices and opinions in case they did not match our search terms. However, this seems less likely given our extended second search round using search terms based on the results of our first search. Lastly, despite our detailed evaluation, we could have overlooked some recommendations or opinions of the CHMP in the QOs, QAs, and SAs, since this part of our research was qualitative by nature.

In conclusion, our study shows that, despite the current pace of technological innovation, the use of RMTs in the regulatory context is still relatively limited. In the absence of formal European guidance on the use of mHealth technologies, our study provides insight in the main recommendations and attention points of the CHMP. These include relevance of the novel endpoint for the indication of interest; validation with current golden standard and legacy endpoints, including those endpoints that matter most to patients; sensitivity, specificity, accuracy, and precision of the novel technology; good compliance and acceptability; and guarantee of optimal data security, and privacy. The development of clear guidance for the use of mHealth technologies in registration trials might promote the development of novel improved endpoints and improve ultimately data quality and regulatory decision making.

## Data Availability Statement

The data presented in this article are not readily available because SAs and QAs contain confidential information that cannot be shared publicly. Requests to access the datasets should be directed to Anna M. G. Pasmooij, am.pasmooij@cbg-meb.nl.

## Author Contributions

MD collected the data and drafted the work. MD and AP were involved in the interpretation of the data. PS and AP revised the manuscript critically. All authors contributed substantially to the conception and design of the work and read and approved the final version of the manuscript.

## Conflict of Interest

The authors declare that the research was conducted in the absence of any commercial or financial relationships that could be construed as a potential conflict of interest.
